# Polymer mesh scaffold combined with cell-derived ECM for osteogenesis of human mesenchymal stem cells

**DOI:** 10.1186/s40824-016-0055-5

**Published:** 2016-04-07

**Authors:** Yong Kwan Noh, Ping Du, In Gul Kim, Jaehoon Ko, Seong Who Kim, Kwideok Park

**Affiliations:** Center for Biomaterials, Korea Institute of Science and Technology, Seoul, 02792 Republic of Korea; Department of Biomedical Science, Kyung Hee University, Seoul, 02447 Republic of Korea; Department of Technical application, Korea Institute of Industrial Technology, Gyeonggi, 31056 Republic of Korea; Department of Biochemistry and Molecular Biology, Asan Medical Center, University of Ulsan College of Medicine, Seoul, 05505 Republic of Korea; Department of Biomedical engineering, University of Science and Technology, Daejeon, 34113 Republic of Korea

**Keywords:** Osteogenesis, Cell-derived extracellular matrix (CDM), Polymer mesh scaffold, Umbilical cord blood-derived mesenchymal stem cells (UCB-MSCs), Microenvironment

## Abstract

**Background:**

Tissue-engineered scaffold should mimic the structure and biological function of the extracellular matrix and have mechanically supportive properties for tissue regeneration. In this study, we utilized a PLGA/PLA mesh scaffold, coated with cell-derived extracellular matrix (CDM) and assessed its potential as an osteogenic microenvironment for human umbilical cord blood-derived mesenchymal stem cells (UCB-MSCs). CDM was obtained by decellularization of in vitro-cultured type I collagen overexpressing (Col I -293 T-DK) cells. Test groups are mesh itself (control), fibronectin-coated (FN-mesh), and CDM-coated mesh scaffold (CDM-mesh). CDM was then solubilized and used for scaffold coating.

**Results:**

CDM was successfully collected and applied to mesh scaffolds. The presence of CDM was confirmed via SEM and FN immunofluorescence. After then, UCB-MSCs were seeded into the scaffolds and subjected to the induction of osteogenic differentiation for 21 days in vitro. We found that the seeded cells were viable and have better proliferation activity on CDM-mesh scaffold. In addition, when osteogenic differentiation of UCB-MSCs was examined for up to 21 days, alkaline phosphatase (ALP) activity and osteogenic marker (COL I, ALP, osteocalcin, bone sialoprotein) expression were significantly improved with UCB-MSCs when cultured in the CDM-mesh scaffold compared to the control and FN-mesh.

**Conclusion:**

Polymer mesh scaffold incorporated with CDM can provide UCB-MSCs with a better microenvironment for osteogenesis in vitro.

## Background

Bone tissue engineering has an ultimate goal to regenerate damaged or lost bone tissue via osteoconductive and/or osteoinductive scaffolds [1]. The 3D polymer scaffolds is supposed to provide appropriate microenvironments to support stem cells adhesion, growth and differentiation, making them suitable for a new bone formation [2]. To do this, polymer scaffolds have been combined with biomaterials derived from natural sources. Examples are collagen [3], gelatin [4], fibrin [5], silk fibroin, keratin [6], and others [7]. In addition, extracellular matrix (ECM) is a complex network of a variety of proteins, proteoglycans, and other macromolecules, where it can provide structural and biochemical support to the surrounding cells [8, 9]. It is well recognized that ECM microenvironments are critical to support cell adhesion, migration, proliferation, and differentiation [10]. Therefore, many studies have also utilized ECM as a valuable resource in tissue engineering [11]. Specifically, ECM obtained from in vitro cultured cells has been studied as a source of bone tissue engineering [12, 13]. Cell-derived extracellular matrix (CDM) promotes osteogenic differentiation of preosteoblasts and bone marrow mesenchymal stromal cells, respectively [14]. In addition CDMs are obtained from various cell types and their positive effects are investigated on the multi-lineage differentiation of human mesenchymal stromal cells [15].

However few studies have examined the effect of cell-derived ECM in combination with an engineered 3D scaffold. From this perspective, we have developed a new platform, composed of a biodegradable PLGA/PLA mesh scaffold, functionalized with bioactive CDM derived from type I collagen overexpressing (Col I -293 T-DK) cells. Our hypothesis is that CDM coated polymer mesh scaffold can represent 3D microenvironment suitable for MSCs adhesion, proliferation, and osteogenic differentiation. In this work, we selected umbilical cord blood-derived mesenchymal stem cells (UCB-MSCs) as a MSCs source. Like other MSCs, UCB-MSCs possess a high proliferation rate for in vitro expansion and have multi-potency capable of differentiating into osteogenic, chondrogenic, and adipogenic lineage [16, 17]. In our study, we found out a significant improvement of osteogenesis of UCB-MSCs on CDM-treated mesh scaffold.

## Methods

### Preparation of PLGA/PLA mesh scaffolds

Poly (L-lactide-co-glycolide) (PLGA; lactic to glycolic acid molar ratio, 50:50) and poly (L-lactide) (PLA) was purchased from EVONIK. PLGA and PLA fibers, 2–2.5 mm in length, were prepared by using a rotary cutter and their nonwovens were produced via modified wet-laid process. PLGA and PLA fibers were mixed in an aqueous solution with a dispersing agent (1 wt.% Pluronic F127; Sigma-Aldrich) and randomly laid on a wire mesh to filter the liquid. The formed web was subsequently processed through a thermal bonding, in which the web was transferred to a heater and cured at 170 °C for 5 min. The resulting mesh was cut into sheets (4 × 4 × 4 mm, L × W × H) and they were sterilized by soaking in 100 % ethanol under ultraviolet (UV) light.

### Cell-derived matrix (CDM) preparation

Collagen type I-overexpression cell line (Col I-293 T-DK) was cultured at the density of 1.3 × 10^4^ cells/cm^2^ in a 100 mm diameter petri-dish for 4 days in the Dulbecco’s Modified Eagle’s Medium (DMEM) supplemented with 10 % fetal bovine serum (FBS) and 100 U/mL penicillin and 100 μg/mL streptomycin (P/S). At the time of confluence, the cell culture plates were washed twice with phosphate buffered saline (PBS), incubated briefly in a detergent solution containing 0.25 % Triton X-100 and 10 mM NH_4_OH (Sigma-Aldrich) at 37 °C, and then subjected to the treatment of 50 U/mL DNase I and 2.5 μL/mL RNase A (Invitrogen) for 1 h at 37 °C. After the decellularization process, the specimens were washed with PBS thoroughly and stored at 4 °C before use.

### CDM characterization: protein and DNA content

For CDM analysis, DNA was examined from the organic phase using Trizol® reagent (Invitrogen). 0.3 mL of 100 % EtOH was added to isolate the DNA from each sample and after centrifugation the supernatant was collected. The supernatant was washed twice with 0.1 M sodium citrate in 10 % EtOH, then centrifuged at 2000 g for 5 min at 4 °C. The samples were resuspended in 2 mL of 75 % ethanol and centrifuged again. The pellets were dissolved in 300 μL of 8 mM NaOH and subsequently quantified using a NanoDrop ND 1000 Spectrophotometer (Thermo Fisher Scientific). In addition, BCA protein assay kit (23250, Thermo Scientific) was used according to the manufacturer’s instructions to assess the total protein amount of CDM.

### Preparation of CDM-coated mesh scaffolds

After the decellularization, CDM was harvested by gentle pipetting, transferred to 50 mL tubes, and vigorously agitated using a homogenizer (HG-3000, SMT, Japan) until a homogeneous aqueous phase was formed. The polymer mesh scaffolds were then immersed into the CDM suspension solution with a mild agitation and incubated for 24 h. The CDM-coated mesh scaffolds were then freeze-dried overnight. Fibronectin (FN; BD Biosciences)-coated mesh scaffolds were also prepared by soaking the scaffolds in FN solution (50 μg/mL in distilled water) at 37 °C for 1 h. The FN-coated scaffolds were then rinsed with distilled water and freeze-dried. The surface morphology of the FN- and CDM-coated mesh scaffolds was observed via scanning electron microscopy (SEM; Phenom G2 Pro Desktop). In addition, the distribution of the CDM in the mesh scaffolds was visualized via immunofluorescence staining of fibronectin using mouse monoclonal antibody (SC-8422; Santa Cruz Biotechnology) and Alex Fluor 488-conjugated secondary antibody (goat anti-mouse IgG; Invitrogen), respectively.

### In vitro culture of UCB-MSCs and proliferation assay

Human umbilical cord blood mesenchymal stem cells (UCB-MSCs) were kindly provided by MEDIPOST Co (Seoul, Korea). UCB-MSCs were cultured in Minimum Essential Medium alpha medium (α-MEM) supplemented with 10 FBS and 1 % P/S. Cells were seeded at 5 × 10^5^ cells in culture flasks and maintained at 37 °C in a humidified 5 % CO_2_ atmosphere with a medium change twice a week. Passage 9 UCB-MSCs were used throughout the experiments. Mesh scaffolds were transferred into non-adherent 24-well tissue culture plates, onto which UCB-MSCs were slowly inoculated at a density of 5 × 10^4^ cells per scaffold. Cells were allowed to adhere for 3 h and cultured in a growth medium for up to 5 days. Three different test groups (*n* = 3, per group) were prepared: 1) plain mesh (control), 2) fibronectin-coated mesh (FN-mesh), and 3) CDM-coated mesh (CDM-mesh). Cell proliferation was evaluated on 2^nd^ and 5^th^ day of culture using CCK-8 assay (Dojindo, Japan). Aliquots from each sample (100 μL) were transferred into a 96-well plate and measured for the absorbance at a wavelength of 450 nm using a Multiskan microplate reader (Thermo Scientific).

### Osteogenic differentiation of UCB-MSCs

Osteogenic differentiation of UCB-MSCs was induced in the presence of osteogenic supplements such as 10 % FBS, 1 % P/S, 50 μg/mL ascorbic acid, 0.01 M glycerol-2-phosphate, 50 ng/mL bone morphogenetic protein (BMP)-2 and 100 nM dexamethasone for 1 and 3 weeks, respectively. Medium was changed every 2 or 3 day.

### Alkaline phosphatase (ALP) activity

ALP activity of each group (*n* = 4 per group) after osteogenic induction for 3 weeks was analyzed using a Lab Assay ALP kit (Wako Pure Chemicals, Japan). Samples were incubated in the lysis buffer (0.1 % Triton X-100 in PBS) for 30 min at 37 °C. 50 mL of the lysis solution was added to 2 mg/mL of p-nitrophenyl phosphate (Sigma 104 tablet) in 0.1 M Tris–HCl buffer (pH 8.5). The absorbance was measured at 405 nm and normalized to the total amount of proteins in each sample lysate, which was assessed via BCA assay (Thermo Scientific).

### Quantitative real-time polymerase chain reaction (q-PCR)

Gene expression of osteogenic markers, such as bone sialoprotein (BSP), collagen type I (Col I), ALP, and osteocalcin (OC) was analyzed via quantitative real-time PCR. Total RNA was isolated using a Trizol® reagent (Invitrogen) extraction method. The extracted samples were subsequently quantified using a NanoDrop ND1000 Spectrophotometer (Thermo Fisher Scientific). cDNA synthesis was performed using a Maxime RT premix kit (Intron). All polymerase chain reactions were carried out using ABI Prism 7500 (Applied Biosystems) and gene expression level was quantified using SYBR Green (RR420A, TaKaRa). Relative gene expression level was calculated by the delta delta Ct method. The primer sequences of the target genes are as follows BSP: CAACCACCCTCTTCACCACT (forward) and GATCTTCTGGGGTGGTCTCA (reverse); ALP: ATGGGATGGGTGTT CCTACA (forward) and GTCTTAGAGAGGGCGACGTG (reverse); Col I: CAAGAACCC CAAGGACAAGA (forward) and GAATCCATCGGTCATGCTCT (reverse); OC: CCAGTT CTGCTCCTCTCCAG (forward) and GCCCACAGATTCCTCTTCTG (reverse) Housekeeping gene is glyceraldehyde-3-phosphate dehydrogenase (GAPDH): GGGCTCTCCAGAACATCATC (forward) and TTCTAGACGGCAGGTCAGGT (reverse).

### Histological analysis

Harvested samples at 1 and 3 weeks were fixed in 4 % paraformaldehyde for 24 h, dehydrated, embedded in paraffin wax, and cut into 10 μm thickness. Those thin sections (*n* = 4 per group) were then subjected to Alizarin Red, ALP and *von kossa* staining, respectively.

### Statistical analysis

Data are expressed as mean ± standard deviation. Statistical significance is determined via one-way analysis of variance (ANOVA) with a posthoc, Bonferroni’s multiple comparison test (GraphPad Prism 5, La Jolla, CA). Statistical significance is marked as * (*p* < 0.05), ** (*p* < 0.01), or *** (*p* < 0.001).

## Results & discussion

### Characterization of CDM

**Fig. 1:**
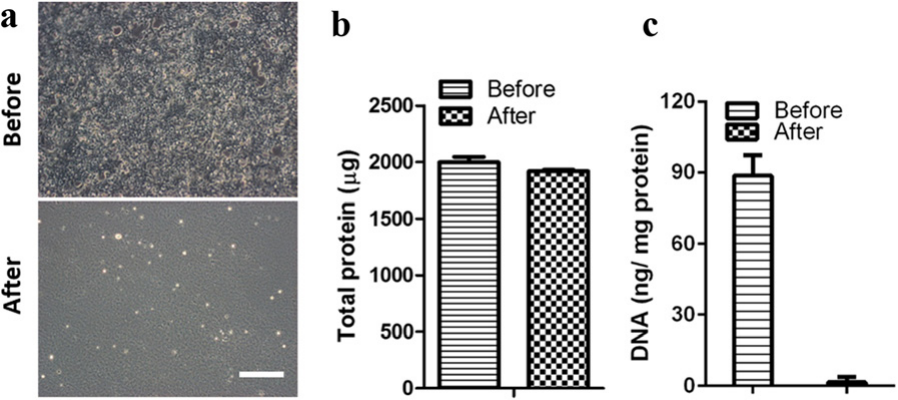
Preparation of CDM and characterization. **a** Cell morphology before decellularization (top) and after decellularization (bottom). **b** Total protein amount before and after decellularization was quantified by BCA assay. **c** DNA content of CDM was also compared before and after decellularization. The scale bar is 200 μm

A confluent cell layer of Col I-293 T-DK cell line on petri-dish was observed under phase contrast microscope (Fig. 1a, top). After decellularization, it was clear that the original cellular morphology were completely removed (Fig. 1a, bottom). The resulting extracellular matrix (ECM) layer is named cell-derived extracellular matrix (CDM). This decellularization procedure is supposed to get rid of cellular components, specifically nucleic and cytosolic ones while retaining bioactive extracellular compositions [18, 19]. Total protein content and DNA amount before and after decellularization were examined, respectively and compared with each other. BCA assay found out that total protein amount of CDM is 1918 μg/per dish, and there is little difference compared to that before decellularization (1996 μg) (Fig. 1b). In addition, it was obvious that cellular DNA was almost completely removed by current decellularization; the amount of DNA (1.5 ng DNA/mg) in CDM was less than 2 % that of the cells (88.61 ng DNA/mg) before decellularization (Fig. 1c). Current DNA amount seems to be negligible, based on the report by Crapo and Gilbert; less than 50 ng of double stranded DNA per mg of ECM should be a minimum criteria regarding the procedure of decellularization [20]. These results demonstrate that our decellularization procedure is effective in clearing cellular DNA while preserving the extracellular macromolecules to a large extent.

### Surface analysis of CDM-coated mesh scaffold

**Fig. 2:**
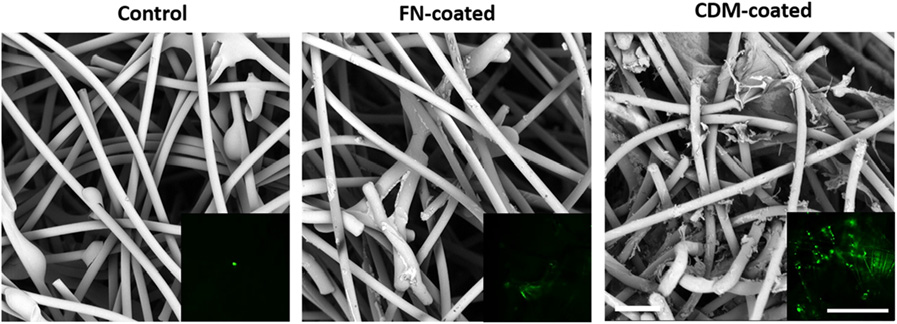
Surface characterization of mesh scaffolds. The surface morphology of differently treated mesh scaffolds (control, FN-coated, and CDM-coated) is observed via SEM. The scale bar is 500 μm. Insets exhibit the distribution of fibronectin (green) in the mesh scaffolds. The scale bar is 200 μm

To confirm whether CDM is successfully coated on the mesh scaffolds, they were examined using SEM and immunofluorescence staining against FN. The SEM images of mesh scaffold without coating exhibited smooth and randomly aligned fibers whereas both CDM-coated and FN-coated group showed the presence of coating material on the microfibers (Fig. 2); FN-coated one shows a thin layer of matrix coating on the microfibers whereas CDM-coated one holds more abundant matrix moieties in the mesh fibers. More specifically, the ECM on the CDM-coated mesh adheres to the microfibers and it also occupies the interstices in the mesh scaffold, contrasted o the ECM on the FN-mesh scaffold. The presence of ECM on mesh scaffold is further confirmed by FN immunostaining, as FN is one of the most important components of CDM [21]. Mesh scaffold alone shows no positive signals of FN. Compared to the FN-coated microfiber, CDM-coated one exhibited much better FN positive signals that homogeneously distributed through mesh scaffold (Fig. 2, Inset). Current data demonstrate that CDM coating is effective and rather homogeneous through the mesh scaffold.

### UCB-MSCs viability and proliferation

**Fig. 3:**
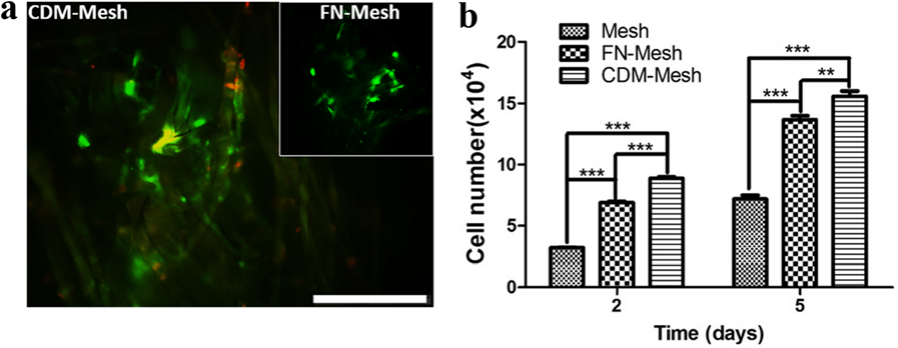
UCB-MSCs viability and proliferation in the mesh scaffold. **a** The viability of UCB-MSCs cultured on various mesh scaffolds was examined via Live & Dead staining at 2 day. The scale is 200 μm. **b** UCB-MSCs proliferation cultured on control, FN-coated, and CDM-coated mesh scaffold is measured at 2 and 5 day, respectively via CCK-8 assay (*n* = 3; **p < 0.01, ***p < 0.001)

To evaluate the capacity of the CDM-coated mesh scaffolds for cell attachment and growth, we cultured UCB-MSCs and examined cell viability via Live & Dead staining after 2 days post-seeding. Representative images of UCB-MSCs exhibited that they are well attached to both CDM- and FN-coated mesh scaffold and the cells are viable (stained in green) in these scaffolds (Fig. 3a). As the proliferation of UCB-MSCs is further examined for up to 5 days via CCK-8 assay, the cell number continuously increased with time on all the mesh scaffolds. However, CDM-mesh was a significantly better on UCB-MSCs proliferation than FN-mesh and plain mesh (Fig. 3b). It can be explained that, comprised of various ECM macromolecules [22, 23], CDM not only provides abundant cell-binding motifs but also plays a certain role in promoting cell proliferation [22].

### ALP activity and gene expression of osteogenic markers

**Fig. 4:**
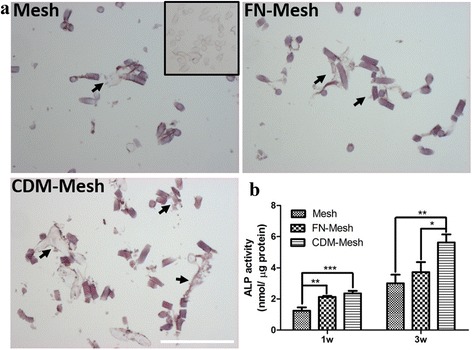
Alkaline phosphatase (ALP) staining and ALP activity measurement. **a** UCB-MSCs cultured in osteogenic medium for 3 weeks were subjected to ALP staining. Insets show the background staining. The scale is 200 μm. **b** When the ALP activity of UCB-MSCs is also measured, that of CDM-coated mesh scaffold is significantly higher than the other groups at 3 week (*p < 0.05, **p < 0.01, ***p < 0.001)

**Fig. 5:**
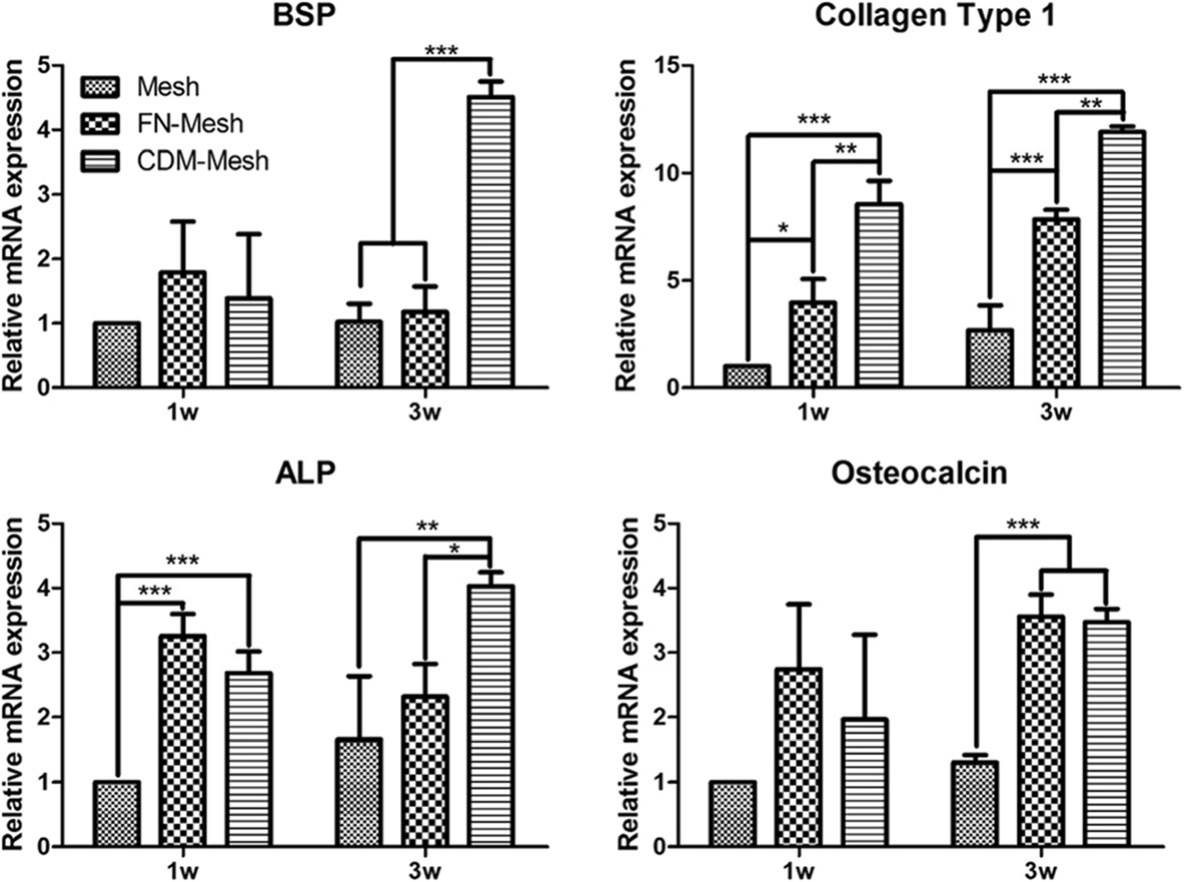
Q-PCR analysis of osteogenic gene expression. Gene expression of BSP, Col I, ALP, and OC was examined via Q-PCR after 1 and 3 weeks of culture in the mesh scaffolds. Gene expression levels of FN and CDM-mesh group were normalized to that of the control (plain mesh) (*p < 0.05, **p < 0.01, ***p < 0.001)

The effect of CDM-coated mesh scaffolds in facilitating MSCs differentiation is examined by culturing UCB-MSCs in osteogenic medium for 1 and 3 weeks, respectively. When the samples were subjected to ALP staining at 3 weeks, CDM-coated mesh was much more positive in ALP activity (Fig. 4a). Although ALP activity increased with time for all the groups, a significantly higher level of ALP activity was observed at both 1 and 3 weeks from the cells cultured on the CDM-coated mesh than those cultured on FN-mesh or control (Fig. 4b). To further examine UCB-MSCs osteogenic differentiation in gene level, Col I, ALP, OC, and BSP are used as osteogenic markers (Fig. 5). Expression levels of both ALP and BSP are relatively higher at 1 week in FN-mesh and CDM-mesh group compared to that of plain mesh. However, the expression level of BSP and ALP was significantly up-regulated at 3 weeks only in CDM-mesh, more than 3-fold greater than that of the other two groups. Similarly, the expression of Col I and OC was also up-regulated in the CDM-coated mesh with time. These results indicate that CDM can promote UCB-MSCs osteogenic differentiation in 3D environment and are better than FN.

### Histological analysis

**Fig. 6:**
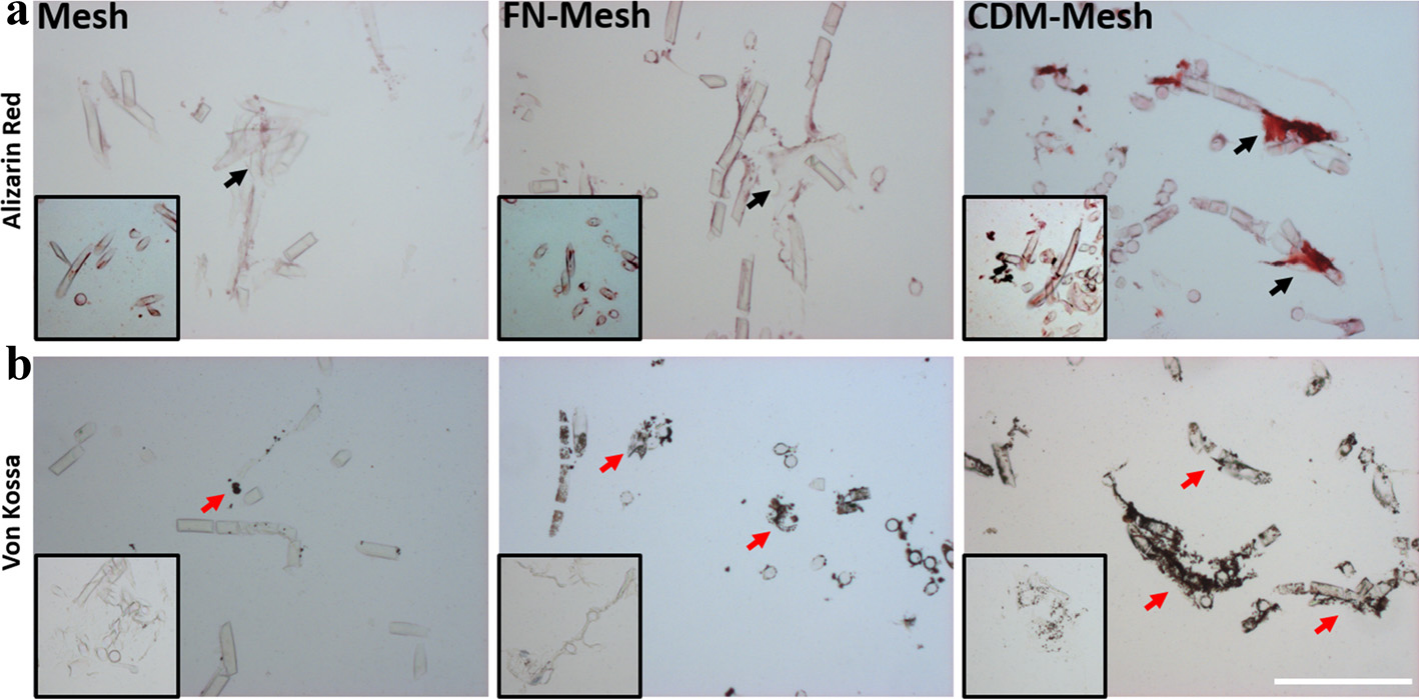
Histological analysis. **a** UCB-MSCs cultured on the mesh scaffolds were visualized by Alizarin red staining and **b** *von kossa* staining at 3 week, respectively. Mineralized matrix deposition was observed for both stainings, particularly more intense in the CDM-mesh scaffold. Insets are the samples stained at 1 week. The scale is 200 μm

Osteogenic differentiation of UCB-MSCs on various mesh scaffolds was also analyzed by Alizarin red and *von kossa* staining, respectively. While Alizarin red staining exhibited a sign of calcium deposition (red color) in the CDM-mesh, both FN-mesh and control presented few positive signals (Fig. 6a). *Von kossa* staining also confirms much better calcium accumulation (black color) with the CDM-mesh, and some positive signals were found on the FN-mesh at 3 week (Fig. 6b). Insets exhibit the corresponding staining of each group at 1 week.

## Conclusions

In this study, CDM obtained from in vitro cultured Col I-overexpression cells was collected and successfully coated onto 3D mesh scaffold. CDM provides a much better microenvironment for UCB-MSCs adhesion and proliferation than FN. More importantly, CDM-coated mesh scaffold supports UCB-MSCs osteogenic differentiation, much better than FN-coated one as indicated by protein and gene expression as well as by histological staining. However, the related cellular and molecular mechanism behind how CDM up-regulates the osteogenic differentiation of UCB-MSCs warrants further investigation. In summary, combination of CDM and polymer mesh scaffold can produce a biomimetic 3D microenvironment and make it a suitable platform for further investigation of stem cell culture and differentiation.
